# Management of a Traumatic Flap Dislocation Seven Years after LASIK

**DOI:** 10.1155/2011/514780

**Published:** 2011-12-27

**Authors:** Majid Moshirfar, Erik Anderson, Nathan Taylor, Maylon Hsu

**Affiliations:** ^1^Department of Ophthalmology and Visual Sciences, John A. Moran Eye Center, University of Utah, Salt Lake City, UT 84132, USA; ^2^University of Illinois College of Medicine, Chicago, IL 60612, USA

## Abstract

Seven years after uneventful laser in situ keratomileusis (LASIK), a 48-year-old woman presented one week after being hit with an iron cord with blurry vision, pain, and irritation. The injury resulted in traumatic flap dislocation, epithelial ingrowth, and macrostriae. Following epithelial removal, the flap was refloated and repositioned. Nine interrupted sutures were used to secure the flap. Three-weeks after surgery with no sutures remaining, the epithelial ingrowth and macrostriae had resolved with a visual acuity of 20/20.

## 1. Background

Flap dislocations are rare complications of laser-assisted in situ keratomileusis (LASIK). A study of army service members who had refractive surgery found the incidence of flap dislocation to be 0.085% (1/1174 eyes). The Wills Eye Institute reported a higher incidence of 2.75% (3/109 patients) in referrals of patients dissatisfied with LASIK.

Most dislocations occur in the early postoperative period as a result of mechanical disruption such as forceful blinking or eye rubbing. However, Schmack et al. showed that LASIK flaps may be lifted without complications up to 8 years, 5 months after initial surgery. We present the following case to describe the surgical management of late-onset flap dislocation.

## 2. Case Report

A 48-year-old woman was referred to us for management of a traumatic LASIK flap dislocation one week after being hit with an iron cord. The patient was seen one day after injury by an outside ophthalmologist and managed with a bandage contact lens and topical antibiotics. On presentation, the patient reported blurry vision, foreign body sensation, pain, and irritation. Associated symptoms included nausea and headache.

The patient had undergone myopic LASIK 7 years, 8 months earlier with a pre-LASIK manifest refraction of −1.75 and −2.00 sphere in the right and left eyes, respectively. Her postoperative course was uneventful as she recovered to best corrected visual acuity of 20/20 in both eyes.

**Figure 1:**
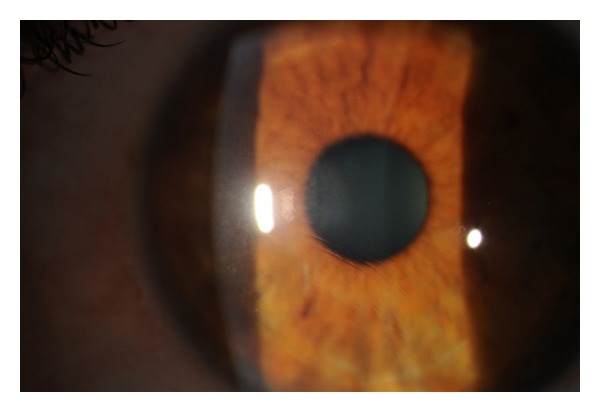
Slit lamp photograph of the injured eye five days after flap dislocation showing several macrostriae approaching the inferior visual axis.

One week after injury, visual acuity was 20/20 in the right eye and 20/40 in the left eye. Ophthalmologic examination revealed a corneal flap displaced 0.25 mm inward from 4 o'clock to 9 o'clock with macrostriae approaching the visual axis inferior temporally (Figure 1). In addition, there was early epithelial ingrowth at the flap edge with a widened gutter margin in the affected area.

Because of the location and extent of the dislocation, the patient was taken to the operating room for flap lifting and repositioning the following day. After a lid block was performed, the inferior half of the flap was lifted and reflected superiorly. All epithelial ingrowth from the posterior surface of the flap was thoroughly removed using weck cells, and the macrostriae were smoothed. The flap was then refloated and repositioned with balance salt solution and a LASIK canula. Nine interrupted 10-0 nylon sutures were used to secure the flap. The knots were rotated away from the flap but not buried to avoid stress on the flap. A bandage contact lens (BCL) was placed and removed three weeks postoperatively. The patient was started on prednisolone 1% every two hours with tapering over the following three weeks. In addition, gatifloxacin 0.5% was used four times a day for one week and then twice a day until the BCTL was removed.

**Figure 2:**
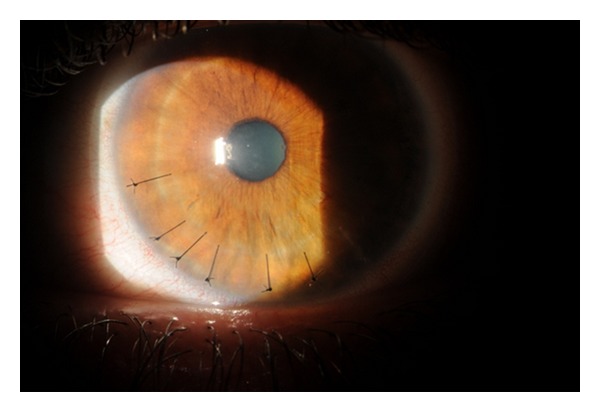
Slit lamp photograph of the injured eye one week after surgical correction with 3 sutures removed.

**Figure 3:**
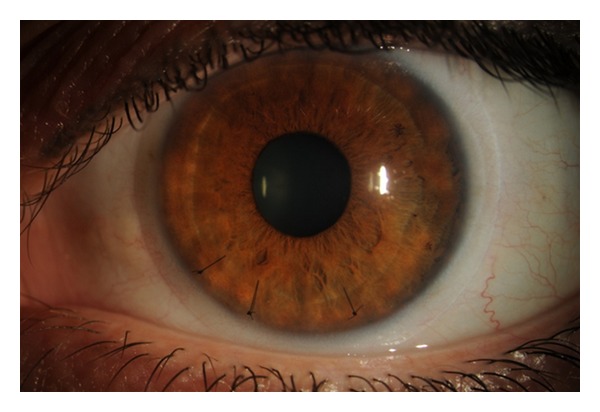
Slit lamp photograph of the injured eye two weeks after surgical correction with only 3 of the original 9 sutures remaining under a bandage contact lens.

**Figure 4:**
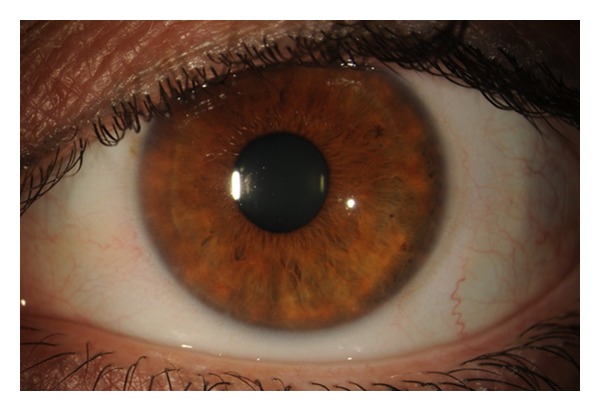
Slit lamp photograph of the injured eye four weeks after surgical correction with all sutures removed with no visible striae.

Five days after surgery, visual acuity was measured at 20/70 in the left eye. There were mild Descemet's folds and cornea edema, but no flap striae. Two to three sutures were removed each visit, beginning one week after surgery in an alternating fashion (Figures 2 and 3). Three weeks after surgery with no sutures remaining, the vision improved to 20/20 and the BCL was removed. The patient was continued on artificial tears every 30 to 45 minutes to prevent epithelial ingrowth. In addition, superior and inferior punctal plugs were placed. At the most recent followup visit (Figure 4), all sutures have been removed with no visible striae.

## 3. Comment

There are numerous case reports and small case series of late-onset flap dislocations. Holt et al. recently reported the longest documented interval from LASIK surgery to traumatic flap dislocation at 14 years after LASIK.

In a brief literature search, we found 11 cases that presented at least 24 months after LASIK [1–9]. We found the majority of dislocations were caused by minor shearing force trauma such as a fingernail injury. The average age of the 11 cases was 32 years old with gender distribution of 7 men to 4 women. One patient did present asymptomatically from an anteroposterior directed trauma as a result of a deployed airbag [5].

These case reports show that the flap-stromal bed interface remains susceptible to dislocation due to wound healing mainly at the edge flap and not at the flap interface. Using electron microscopy, Rumelt et al. showed that collagen fibrils on both sides of the incision were well aligned, with an identical diameter (27 to 35 nm) to fibrils on other parts of the cornea. In contrast, there were an absence of bridging collagen fibrils and cells between the flap undersurface and the stromal bed confirming the clinically known lack of wound repair at the interface [10]. Patients should be educated about the risk of flap dislocation and urged to wear eye protection when necessary. Many of the reported flap dislocations could have been prevented with the use of protective eyewear.

Even though the delayed presentation did not seem to affect the outcome, the recognition of refractive surgery complications is important. Thus, any patient with an ocular injury should have a thorough refractive surgical history taken.

Conventional management of a dislocated flap uses medical eye drops and a bandage contact lens. For larger areas of dislocation, surgical management with lifting and repositioning the flap is preferred. In our case, the flap was secured with nine sutures with gradual suture removal. This method ensures that a persistent fold does not form and decreases the chance of reoccurrence. The use of sterile water has also been reported to promote swelling and decrease striae [8]. Epithelial ingrowth is the most common reported complication and requires complete scrapping and removal to prevent reoccurrence. Fortunately, proper management can result in a return of baseline vision for most dislocations.
